# Investigation of Commensal *Escherichia coli* Populations of Cormorant Hatchlings in the Absence of Anthropogenic Impacts in Remote Areas of West Mongolia

**DOI:** 10.3390/microorganisms9020372

**Published:** 2021-02-12

**Authors:** Muhammad Moman Khan, Rafal Kolenda, Peter Schierack, Jörg Weinreich, Stefan Rödiger, Jakob Schierack, Michael Stubbe, Davaa Lkhagvasuren, Sebastian Guenther, Katharina Schaufler

**Affiliations:** 1 Institute of Biotechnology, Faculty of Environment and Natural Sciences, Brandenburg University of Technology Cottbus-Senftenberg, 01968 Senftenberg, Germany; khanm@b-tu.de (M.M.K.); peter.schierack@b-tu.de (P.S.); joerg.weinreich@b-tu.de (J.W.); stefan.roediger@b-tu.de (S.R.); 2 Department of Biochemistry and Molecular Biology, Faculty of Veterinary Medicine, University of Environmental and Life Sciences, 50-375 Wrocław, Poland; rafal.kolenda@upwr.edu.pl; 3 Faculty of Health Sciences, Joint Faculty of the Brandenburg University of Technology Cottbus-Senftenberg, The Brandenburg Medical School Theodor Fontane and the University of Potsdam, 01968 Senftenberg, Germany; 4 Lessing Gymnasium, 02977 Hoyerswerda, Germany; jakobschierack@gmail.com; 5 Gesellschaft für Wildtier- und Jagdforschung e.V., 06099 Halle/Saale, Germany; stubbe@zoologie.uni-halle.de; 6 Department of Biology, School of Arts and Sciences, National University of Mongolia, Ulaanbaatar 14200, Mongolia; lkhagvasuren@num.edu.mn; 7 Pharmaceutical Biology, Institute of Pharmacy, University of Greifswald, 17489 Greifswald, Germany; sebastian.guenther@uni-greifswald.de; 8 Pharmaceutical Microbiology, Institute of Pharmacy, University of Greifswald, 17489 Greifswald, Germany

**Keywords:** adhesion, antibiotic resistance, commensal, cormorants, *E. coli*, virulence

## Abstract

To increase our understanding of bacterial intestinal colonization in animal populations lacking substantial anthropogenic influence we studied the diversity of *E. coli* in cormorants from the pristine West-Mongolian steppe. *E. coli* were isolated from individual birds of two cormorant colonies located on small islands in lakes at least 100 km away from human settlements. Diversity of the isolates was studied using pulsed-field gel electrophoresis (PFGE). 137 isolates of cormorant colony-1 and 75 isolates of cormorant colony-2 resulted in 60 and 33 PFGE types, respectively. Representative strains of each PFGE type were analyzed via PCR in terms of phylogroups and extraintestinal virulence-associated genes (exVAGs). Bacterial adhesion to the chicken intestinal cell line CHIC-8E11 and antimicrobial resistance was also determined. Most isolates belonged to phylogroup B1 (68.3%) followed by B2 and E with B2 harboring the highest total number of exVAGs per isolate. Unexpectedly, a PFGE type with relatively few exVAGs displayed the highest isolation frequency, also showing a high adhesion rate. Comparative analysis of exVAGs to other *E. coli* populations of wildlife origin revealed that the secreted autotransporter toxin encoding *sat* gene was only present in cormorants. Overall, *E. coli* in cormorants maintained a high diversity under minimal anthropogenic influences, which likely enables intestinal colonization.

## 1. Introduction

Bacterial colonization in animal guts starts early on during birth and in continuous stages until the intestinal microbiome reaches a functional, complex and dynamic ecosystem [1]. Depending on the bacterial strains in such microbiological environments, colonization varies and persists over months or years after a niche was developed whereas others may disappear with time even within weeks [2]. Bacterial colonization can be defined as the indefinite presence of a particular population of bacteria without reintroduction of that particular population. Studies on human fecal samples showed that—despite external and internal pressures—they are colonized on average by five different *E. coli* strains [3]. In fecal content of pigs raised in the same house, even 34 different *E. coli* strains were found. Additionally, if five different individuals in a similar environment are taken together, diversity increases as each might have different strains at a particular time showing that intestinal *E. coli* strains strongly vary between individuals. Diversity is highly dynamic; even in piglets from the same environment [4]. It can be concluded that diversity exists among commensal *E. coli* strains and that they may possess different strategies and physiological properties to colonize and inhabit the intestinal environment [3]. Antibiotic resistance may also confer additional competitive advantages to those strains. Finally, all other factors being equal, the strains that are able to grow and survive in these environments ultimately lead to more success [5].

*Escherichia coli* is a metabolically versatile bacterium that colonizes the intestines of mammals and birds [6]. Some *E. coli* strains are pathogenic, causing various intestinal and extraintestinal diseases affecting humans and animals [7]. The species has a variety of virulence-associated factors, which can also be found in healthy hosts rendering colonization advantages such as adhesins, toxins, iron acquisition factors, lipopolysaccharides, polysaccharide capsules and invasins, which are frequently encoded on mobile genetic elements [4]. Commensal *E. coli* may serve as a relevant and representative model for establishing microbial principles in the intestinal microbiome due to its demonstrative features of the whole microflora [8]. Intestinal *E. coli* populations are individual, dynamic, and very complex, but are not well understood. Already published studies show factors such as metabolism, microcin production, virulence-associated genes of extraintestinal pathogenic *E. coli*, antibiotic resistances and other factors facilitate successful bacteria colonization [2,4,5,9]. Research on intestinal *E. coli* strains focusing on diversity, virulence traits, phylogenetic categorization and antibiotic resistance gives vital insights about harboring hosts and the risks for diseases, pathogen transmission, or resistance/endurance to antibiotics [10]. Already published work focused on comparative advantage and/or disadvantage of competitive colonization using mouse models, cell culture models or bioreactors with limited bacterial strains. Additionally, little data is available on the complex dynamics of commensal *E. coli* populations and their colonization in a natural environment devoid of anthropogenic pressures.

The aim of this study was to observe bacterial intestinal colonization parameters and explore the reasons behind dominance or higher prevalence of particular strains in isolated naturally occurring animal populations in the (mostly) absence of anthropogenic and antibiotic selection pressures. The present study focuses on two separate colonies of Great Cormorant (*Phalacrocorax carbo*) hatchlings sampled during bird ringing on small islands of ~200 m^2^ in the middle of two different lakes at least 100 km away from permanent human settlements in Mongolia and 568 km from each other. The lakes were in the middle of the semi desert or desert steppe, where livestock was absent, and the lake was the only source of food and water. The islands were densely populated with cormorant nestlings with a distance of less than one meter between nests. Given the close proximity of the birds and the single source of feeding from only one lake and low anthropogenic influences, we would have expected the *E. coli* diversity to be rather low. Although we assume that *E. coli* harbored by cormorants in remote Mongolia areas are not exposed to antibiotic selection pressure, the animals can ingest and thus harbor antibiotic-resistant *E. coli* due to bird migration in their winter quarters.

Evaluation of the distribution of phylogenetic groups, prevalence of virulence-associated genes and adhesion rates of *E. coli* isolates as well as determination of the sensitivity to antimicrobials increase our understanding and explore possible reasons for the colonization abilities and diversity of commensal *E. coli* strains in remote natural environment lacking substantial anthropogenic influences.

## 2. Materials and Methods

### 2.1. Definitions

Isolates of one PFGE (pulsed-field gel electrophoresis) type did not differ in more than three bands from each other [11]. Extraintestinal virulence-associated genes (exVAGs) are virulence-associated genes which are involved in the *E. coli* pathogenesis of extraintestinal infections including infections caused by avian pathogenic *E. coli* (APEC). exVAGs were also shown to support colonization of *E. coli* in healthy hosts [4].

### 2.2. Study Area

**Figure 1:**
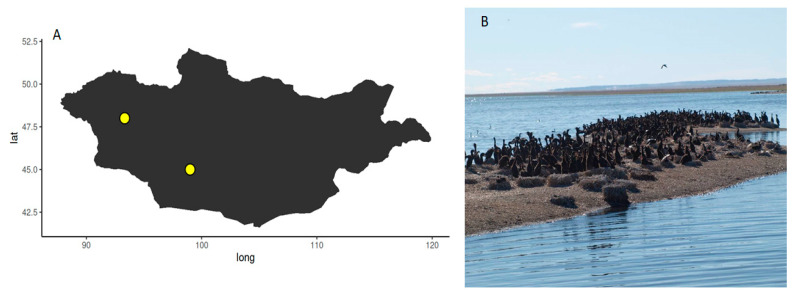
(**A**) Map of Mongolia depicting the sample locations of *E. coli* isolates; (**B**) cormorant colony-1 on an island in the lake Buuntsagaan, Bayankhongor province (N48° E93.19′19.2″).

*E. coli* were sampled on a bird ringing expedition in Mongolia in July 2017 during the Mongolian-German Biological Expedition No. 347, which have been organized since 1962. Cormorant colony-1 was located in the lake Buuntsagaan, Bayankhongor province (N45° E99°), Cormorant colony-2 was located the lake Airag, Uvs province (N48° E93.19′19.2″) as shown in Figure 1. The overall population data for Mongolia are as follows: human density: 1–2 n/km^2^, livestock densities: swine < 1 n/km^2^, cattle 1–5 n/km^2^, small ruminants 5–10 n/km^2^, poultry < 10 n/km^2^. These very low numbers are expected to be even lower in the sampling locations of this study as they are among the least densely human-populated areas in the world and livestock as well as large cities were not present [12].

### 2.3. Collection of Bacterial Isolates

During bird ringing, we sampled hatchlings once and shipped cloacal swabs (MASTASWAB containing Amies Medium with charcoal, Mast Diagnostica GmbH Reinfeld, Germany) to the lab in Senftenberg, Germany. Cloacal swabs were streaked on CHROMagar orientation plates (Mast Diagnostica GmbH, Reinfeld, Germany [13]) and one single pink colored colony from each plate per bird was taken [14,15], and was further confirmed by MALDI-TOF (microflex, Bruker, Billerica, MA, USA). One single *E. coli* colony was then subcultured twice on CHROMagar orientation plates and stored in 20 % glycerol at −80 °C until further processing. All *E. coli* isolates were categorized by macrorestriction analysis to PFGE types using *XbaI* restriction digestion as previously described [9].

### 2.4. Antimicrobial Susceptibility Testing

One isolate of each PFGE type from cormorant colony-1 was tested for antimicrobial resistance against 28 substances/combinations (Table S1) by the agar disk diffusion method according to the Clinical and Laboratory Standards Institute (CLSI) [16].

### 2.5. DNA Preparation, Virulence, and ECoR Genotyping

One isolate of each PFGE type from cormorant colony-1 was investigated for 30 exVAGs also typical for avian pathogenic *E. coli* by multiplex PCR as described before [17,18]. They were classified according to the *E. coli* Reference Collection (ECoR) system [19] by use of a PCR previously described [20,21]. Selection of exVAGs, primers (BioTez, Berlin, Germany) and PCR conditions are based on a previous study [17].

### 2.6. E. coli Adhesion Assay

Chicken intestinal cells CHIC-8E11 (MicroMol GmbH, Karlsruhe, Germany) were grown in 96-well plates (Nunc, Langenselbold, Germany) in Dulbecco’s modified Eagle Medium (DMEM) HAM’S/F-12 (1:1) (Biochrom, Berlin, Germany), supplemented with 10% fetal calf serum (Biochrom, Berlin, Germany), 1% L-Glutamine (Biochrom, Berlin, Germany), and maintained in an atmosphere of 5% CO_2_ at 37 °C [22]. Adhesion assays were performed essentially as previously described [23]. Confluent CHIC-8EII cell monolayers were inoculated with one *E. coli* isolate of each PFGE type from cormorant colony-1 with an infection dose of 62,500 bacteria per mm^2^ of a cell monolayer using a conversion factor of approximately 3 × 10^8^ bacteria/mL/OD_600_. This infection corresponded to a multiplicity of infection (MOI) of 100:1 *E. coli* to host cells. Cells were incubated with *E. coli* for 3 h at 37 °C. A FISH staining method was used for quantification of adherent bacteria on chicken cell line [24]. The plates were washed three times with distilled water. After fixation by formaldehyde (4%), cells were dehydrated with 50 μL of 95% ethanol for 5 min, dried and stored at 4 °C until performing FISH. 40 μL of FISH probe EUB338 Atto647N with a final concentration of 5 ng/μL was added to each well and the plates were incubated at 46 °C for 1 h in a humid chamber [24]. The plates were washed once with a washing buffer and incubated at 48 °C for 10 min in the washing buffer. Nuclei were stained with DAPI (50 μg/mL in distilled water) and washed once with 1× PBS. Finally, plates were analyzed by the Aklides^®^ system (Medipan GmbH, Potsdam, Germany) [17,25].

We classified bacterial adhesion as: (1) low (1–2000 bacteria/mm^2^); (2) medium (2001–4000 bacteria/mm^2^); and (3) highly adherent (more than 4000 bacteria/mm^2^). Assays were done in triplicate wells and were repeated at least three times.

### 2.7. Statistical Analyses

Statistical analysis was carried out using the R statistical software [26]. Gene prevalence was compared with the Chi-squared test of independence implemented in the R package. Figures were generated with the use of ggplot2 package implemented in R software [27]. The data were reordered by hierarchical clustering analysis using complete linkage method and R software [28]. Analysis of gene combinations among the virulence gene pattern of *E. coli* isolates was performed with Microsoft Office Excel 2003 (Microsoft, Redmond, WA, USA).

### 2.8. Ethics Statement

We carried out the sampling of nestlings in Mongolia during bird ringing and the animals were released afterward in accordance with the Ornithological Council’s guidelines on the use of wild birds in research [29]. We conducted sampling in Mongolia with the approval and in cooperation with the National University of Mongolia in Ulaan-Baatar, Mongolia. According to the IUCN Red List of Threatened species, the conservation status of cormorants (*Phalacrocorax carbo*) of this study was of “least concern” (LC).

## 3. Results

### 3.1. Clonal Diversity of E. coli Populations of Two Cormorant Colonies

As a result, of PFGE typing, out of 137 isolates of cormorant colony-1 we defined 60 PFGE types. High diversity in terms of PFGE types was exhibited where 43 PFGE types were represented by single isolate per PFGE type. The most prevalent PFGE type of cormorant colony-1 had 39 isolates. In case of cormorant colony-2 with 75 isolates, we defined 33 PFGE types where 22 PFGE types had only one isolate, the maximum was one PFGE type with 11 isolates (Table 1). Due to the fact that the most prevalent PFGE type was present in cormorant colony-1 along with a high diversity, we decided to investigate this colony further in terms of exVAGs prevalence, phylogroups, adhesion rates and antibiotic resistance.

### 3.2. Virulence-Associated Gene Profiles and Phylogenetic Affiliation

Different exVAGs have been shown to be intestinal bacterial colonization factors and we screened one strain from each of 60 PFGE types of cormorant colony-1 for 30 exVAGs using PCR (Table 2). Overall, the prevalence of exVAGs was low and differed markedly between different PFGE types. Genes associated with large plasmids, occurred in a substantial number only in the case of *traT* (60%), whereas others, including *iss* (10%), *sitep* (1.7%), *iutA* (6.7%), *tsh* (1.7%) and *iucD* (5%), yielded a lower prevalence. *iroN* and *cvi/cva* were not detected in the entire population of *E. coli* in the cormorant colony-1. Additionally, *afa/dra*, *hlyA*, *papC* and *cnf1/2* were also absent in the whole population, every other exVAG was found in at least one isolate, with *fimC* (100%) and *ompA* (100%) being the most prevalent and *ibeA* (5%), *sfa/foc* (3.33%), *pic* (3.33%), *sitep* (1.67%), *tsh* (1.67%) and *tia* (1.67%) with the lowest prevalence (Table 2). There were seven isolates with the lowest number of exVAGs in a single isolate, i.e., five exVAGs. Two isolates were found with 13 exVAGs, which is the maximum number in a single isolate.

Each isolate from a single PFGE type was classified into phylogroups according to the ECoR (*E. coli* Reference Collection) system. Most isolates belonged to the ECoR group B1 (68.3%) followed by B2 (15%) and E (6.7%). Only one isolate belonged to group D and none were identified as ECoR group A. Five isolates could not be assigned to any of the ECoR group due to conflicting information by PCR results. Possible correlations between ECoR groups, virulence-associated genes, and isolation frequencies were also analyzed. On average, ECoR group B2 isolates carried most exVAGs per isolate (11 ± 2.0), followed by isolates of group B1 (7.2 ± 1.8) and E (6.75 ± 1.3). Isolates grouped as undetermined in ECoR groups carried 7.4 ± 1.8 exVAGs per isolate.

### 3.3. Adhesion Assays

Since there is no cormorant cell line available, we used the intestinal chicken cell line CHIC-8E11 and tested one isolate from each PFGE type from cormorant colony-1 for adhesion. After incubation of three hours with CHIC-8E11 monolayer, adhesion of *E. coli* varied strongly between ranging from 169 bacteria/mm^2^ to 10,095 bacteria/mm^2^. Most PFGE types can be categorized as low colonizers (n = 42) with adhesion rates with less than 1000 bacteria/mm^2^ followed by medium colonizers, i.e., 2001–4000 bacteria/mm^2^ (n = 12). Six isolates were found to adhere strongly to CHIC-8E11 cells with more than 4000 bacteria/mm^2^.

### 3.4. exVAG Profiles of E. coli and Relationship between Different Colonization Parameters

**Figure 2:**
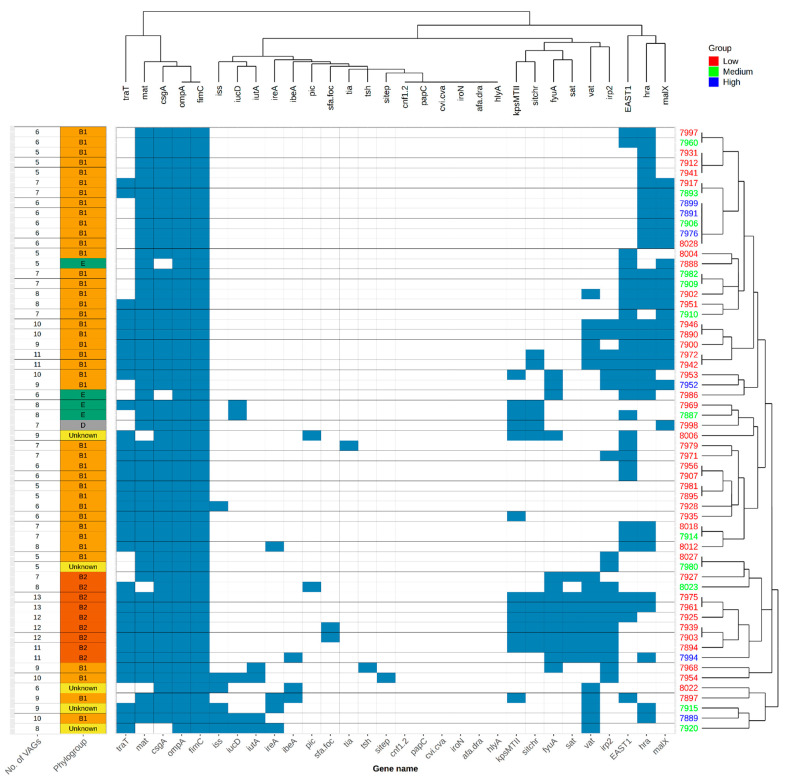
exVAGs profile cluster analysis in association with adhesion rates, phylogroups and number of exVAGs. One strain from each PFGE type was screened for exVAGs, adhesion rates and phylogroup determination.

To analyse the association of exVAGs and the ability of intestinal colonization in the intestine of cormorants, we compared the numbers of exVAGs and adhesion rates via cluster analysis (Figure 2). We found no clustering between exVAGs gene prevalence and adherence rates on the chicken cell line that would point toward a correlation of successful colonization of commensal *E. coli* with the total number of exVAGs present.

**Figure 3:**
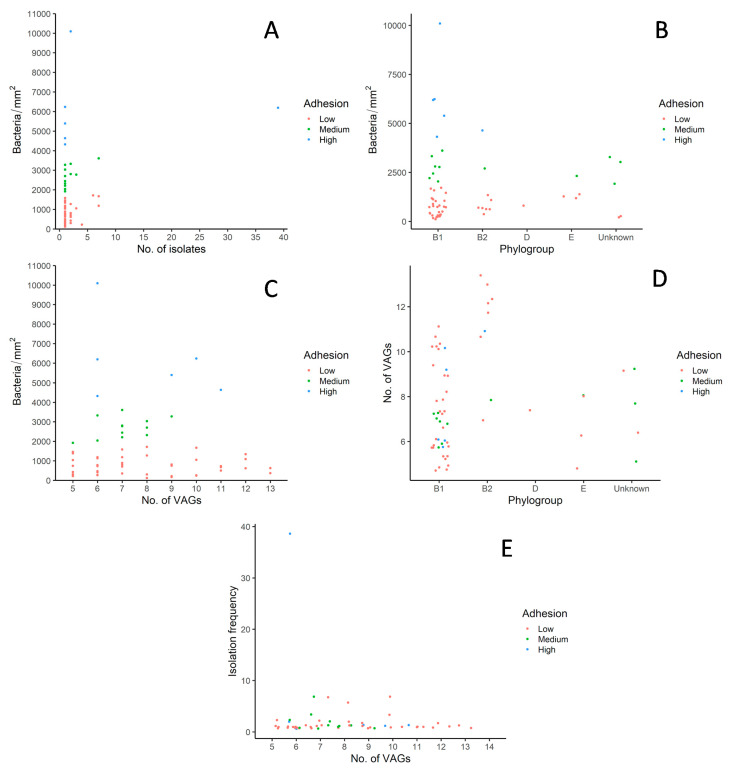
Correlation analysis for colonization parameters of *E. coli* in cormorant colony-1. (**A**) Number of isolates per pulsotype with adhesion rates; (**B**) Phylogroups with adhesion rates; (**C**) Adhesion rates with number of exVAGs; (**D**) Phylogroups with number of exVAGs; (**E**) Number of exVAGs with isolation frequency. Shown are scatterplots with one plot representing one pulsotype.

Further analysis and comparisons were drawn to explore the reasons leading to successful colonization of *E. coli* in the environment devoid of human and antibiotic pressures as shown in Figure 3A–E. Most PFGE types comprising of one to two isolates and exhibiting quite diverse adhesion patterns belonged to phylotype B1 (Figure 3A). Interestingly, the PFGE type with the highest frequency of isolates (39 isolates) was also categorized as B1 and showed a high adhesion rate. Overall, phylogroup B1 displayed the highest number of isolates and exhibited diverse adhesion rates with five, seven and twenty-nine isolates as high, medium, and low adherence to the avian cell line, respectively (Figure 3B). Interestingly, most of the isolates categorized in phylogroup B2 showed low adhesion rates with two exceptions with medium and high adhesion. No conclusion can be drawn for phylogroup D and E due to the low number of isolates. No direct positive or negative correlation can be drawn between the presence of exVAGs and the adhesion rate (Figure 3C). The Figure 3C shows that a higher number of exVAGs does not correlate with a high adhesion rate. The isolate with the highest adhesion rate or around 10,000 bacteria per mm^2^ had only six exVAGs. Whereas the isolates with 12 and 13 exVAGs displayed low adhesion capacities. On average isolates belonging to phylogroup B2 displayed the highest number of exVAGs followed by phylogroup B1, which has a high variation on the average number of exVAGs as presented in Figure 3D. Isolates which could not be categorized in any of the phylogroups labeled as undetermined also show diversity in terms of adhesion rate and number of exVAGs. There is no positive and negative correlation between isolation frequency and the number of exVAGs (Figure 3E). It is worth mentioning that the PFGE type with one of the lowest numbers of exVAGs has the highest isolation frequency with 29 isolates along with a high adhesion rate.

### 3.5. Antimicrobial Susceptibility and Possible Correlations with Adhesion Rates

Resistance to antimicrobial substances affects colonization of intestinal *E. coli* [2,30], and we therefore phenotypically tested one isolate of each PFGE type for antimicrobial resistance using agar disk diffusion assays. In cormorant colony-1, most isolates were susceptible to all antimicrobial substances. Resistances were found to amoxicillin (3 isolates) and ampicillin (9 isolates). Interestingly, one isolate was resistant to seven different substances/combinations, i.e., ampicillin, amoxicillin-clavulanic acid, ampicillin-sulbactam, doxycycline, tetracycline, ticarcillin and trimethoprim.

**Figure 4:**
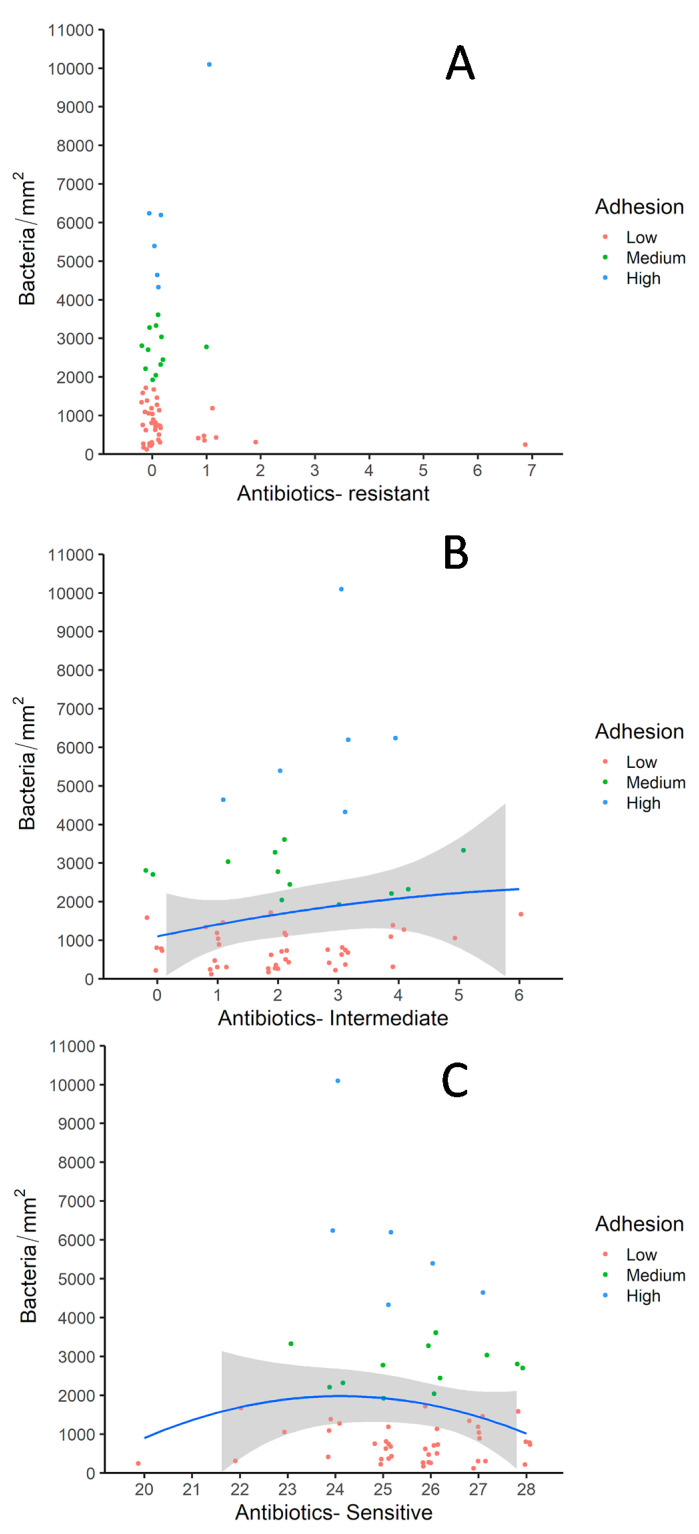
Correlations drawn between antimicrobial susceptibility and adhesion rates of *E. coli* in cormorant colony-1. (**A**) Antimicrobial resistance with adhesion rates; (**B**) Intermediary antimicrobial resistance with adhesion rates; (**C**) Antibiotic sensitivity with adhesion rates. Shown are scatterplots with one plot representing one pulsotype.

Since resistance prevalence was low in the commensal *E. coli* population in this remote area of Mongolia, most of the diverse adherence patterns are observed in isolates with almost no resistance to any of the antibiotics as shown in Figure 4A. The strain which was resistant to seven different antibiotics had a very low adherence rate. In the absence of antibiotic pressure, a significant number of isolates had intermediate resistance (according to CLSI document M100-S23 (M02-A11)) to a diverse group of antibiotics (Figure 4B). Most of the isolates with medium adhesion rates showed intermediate resistance to relatively low numbers of antibiotics. Maximum diversity in terms of adhesion rates was observed in isolates which had intermediary resistance against up to three antibiotics. Overall downward trends in adhesion rates with respect to increased sensitivity against the antibiotics is observed (Figure 4C). Isolates, which tested sensitive to almost all of the 28 antibiotics exhibited low adhesion rates as well.

## 4. Discussion

In contrast to previous works, we studied a bacterial gut population in a pristine animal population originating from an environment with low anthropogenic impact. In this study, we determined the occurrence of intestinal *E. coli* pulsotypes, explored reasons for higher prevalence and the possible correlations between specific bacterial genes with colonization successes of the respective pulsotypes. Diversity, virulence gene profiles, phylogenetic analysis, and antimicrobial resistance provide important information about intestinal *E. coli* populations and possible correlations to bacterial colonization, persistence and survival as well as health, diseases, and disease treatment strategies [9].

Surprisingly, the *E. coli* population was very diverse with up to 60 PFGE types out of 137 isolates of cormorant colony-1 and 33 PFGE types out of 74 isolates from cormorant colony-2. This high diversity was unexpected due to very similar environmental conditions with food from the same source and densely populated bird colonies located on islands. A high diversity within an *E. coli* population seems to be a basic natural phenomenon with possible frequent transmission of clones between individuals [14].

Pearson’s Chi-squared test for count data was carried out to compare the significant prevalence of exVAGs in *E. coli* in cormorants and different hosts and environments i.e., mallard ducks, wild birds and wild mammals from already published studies as shown in Table 2 [2,17]. The same statistical analysis was also carried out to compare significant exVAGs prevalence across the two classes, i.e., birds and mammals.

Secreted autotransporter toxin coding *sat* gene (*p* < 0.05) was absent in *E. coli* of all the other animals except cormorants of Mongolia. The *sat* gene is highly prevalent in Uropathogenic (UPEC) and Diffuse-adhering (DAEC) *E. coli* but its role in infections is dependent on genetic determinants of bacterial pathotypes [31]. The presence of this gene in cormorants of Mongolia indicates such as *E. coli* Nissle 1917 (EcN) that some of the *E. coli* from Mongolia might be closely related to Uropathogeic *E. coli* [31]. The prevalence of toxin encoding gene *east1* and protectin encoding *traT* was significantly higher (*p* < 0.05) in cormorants only when compared to studies of mallard ducks and other wild birds. In contrast, other genes such as *hlyA*, *cvi/cva*, *cnf1/2* and *papC* were absent in *E. coli* from cormorants and other wild birds but had a higher prevalence in mallard ducks. Three iron acquisition genes such as *iroN*, *sitep* and *chuA* are significantly less prevalent (*p* < 0.05) in cormorant colony-1 when compared to *E. coli* populations in other three wildlife populations. When comparing the prevalence of exVAGs in mammals in comparison to wild birds, genes such as *kpsMTII*, *sfa/foc*, *sitchr*, *iroN*, *vat*, *tsh*, *iucD*, *papC*, *iss*, *iutA*, *fyuA*, *malX*, *irp2* and *chuA* were significantly less prevalent in mammals. Only three genes—*csgA*, *ireA* and *tia*—were present more often in mammals.

Phylotyping according to the ECoR system provides a simple tool that allows estimation of pathogenic features of *E. coli*. It has been proposed that the phylogenetic affiliation indicates a specific type/pathotype of *E. coli* [20,32,33]. Phylogenetically, *E. coli* are assigned to seven major phylogenetic groups, namely A, B1, B2, C, D, E and F [21]. Usually, extraintestinal pathogenic strains belong to groups B2 and D, while isolates belonging to groups A and B1 are more often strictly commensal strains from human intestinal microbiota [20,21,34]. Contrary to our present study, where group B1 was mostly isolated in cormorants, most isolated *E. coli* in mallard ducks were found to belong to group B2 followed by B1 [2]. However, strains from group E and D were found in very low percentages in mallard ducks, which is in line with our study. However, as we used cloacal swabs for this study this might have influenced the outcome of phylotyping and in fecal samples the diversity of *E. coli* might be even higher.

Several studies showed that human and animal extraintestinal pathogenic *E. coli* from groups B2 and D harbored a greater frequency and diversity of virulence traits compared with strains of other phylotypes [33,35], which is in line with our study regarding B2 with most exVAGs per isolate (11 ± 2.0).

Adhesion to host epithelial cells is a well-accepted colonization factor [17]. In our study, most strains’ adhesion rates ranged from low to medium and there was no significant correlation between adhesion and the total number of exVAGs. We conclude that other factors mainly affect colonization in cormorants as underlined by the fact that the PFGE type with the highest isolation frequency [29 isolates] also showed the lowest number of exVAGs but displayed a high adhesion rate. Similarly, a higher number of exVAGs does not correlate with a high adhesion rate and it could be based on the right combination of exVAGs and other factors. The isolate with the highest adhesion rate or around 10,000 bacteria per mm^2^ had only six exVAGs. However, due to the lack of a cormorant bird intestinal cell line, we chose one of the avian cell lines for the definition of *E. coli* adhesion. It is possible that adhesion patterns may be different with the cormorant intestinal cell line [2].

Emergence and persistence of antimicrobial resistance in a natural environment over decades, independent of any further direct human influence and presence of antimicrobial substances is of scientific and social concern [2,10,15]. Conclusively, resistant *E. coli* were not significantly advantaged or disadvantaged in competition with susceptible *E. coli* and resistance to more agents was not detrimental in comparison to single resistant *E. coli* in the context of their colonization abilities. This might help to understand the occurrence of resistant bacteria in animal populations over long time periods and might substantiate the potential risk of the application of antimicrobial substances in the animal production [10]. Since we sampled hatchlings it was obvious that resistant *E. coli* were transmitted from adults to the next generation of animals thus showing substantial colonization capabilities of resistant *E. coli* without substantial environmental influences in Mongolia. The migratory route for these cormorants is unknown however it was shown for other birds that they migrate to India or neighboring southern countries [36]. We assume that cormorants have similar southward migration routes, at least during the harsh winters of Mongolia.

## 5. Conclusions

In conclusion, the *E. coli* populations of great cormorant hatchlings were highly diverse even in the absence of external anthropogenic pressures. As we used cloacal swabs for this study, their diversity might be even higher in fecal samples. We hypothesize that many strains can coexist even in a very homogeneous environment.

## Figures and Tables

**Table 1:** Summary of pulsed-field gel electrophoresis (PFGE) types and number of isolates in each type in cormorant colony 1 and 2.

Number of Isolates Per PFGE Type	1	2	3	4	5	6	7	8	11	39
Cormorant colony-1	43 *	9 *	2	1	0	1	3	0	0	1
Cormorant colony-2	21	5	1	1	2	0	0	2	1	0

* 43 PFGE types had one isolate, 9 PFGE types had two isolates, etc.

**Table 2:** Functions and prevalence percentage of detected extraintestinal virulence-associated genes (exVAGs) in *E. coli* in cormorants, mallard ducks, wild birds, and wild animals. Pearson’s Chi-squared test for count data calculated to compare the significant differences in prevalence.

Genes	Functions	Cormorants n = 60	Mallard Ducks n = 220	Other Wild Birds n = 54	Total Wild Birds n = 334	Wild Mammals n = 187
*kpsMTII*	Adhesin	21.7	14.4	0.0 *	13.2	0.0 *^,+^
*afa/dra*	Adhesin	0.0	0.0	0.0	0.0	0.0
*sfa/foc*	Adhesin	3.3	9.3	9.3	8.4	2.1 ^+^
*pic*	Miscellaneous	3.3	9.6	14.8	9.3	14.4 *
*hra*	Adhesin	53.3	17.8 *	40.7	28.4	34.8 *
*hlyA*	Toxin	0.0	8.7 *	0.0	5.7	3.7
*ibeA*	Invasin	5.0	15.2	31.5 *	16.2	16.0 *
*traT* ^a^	Protectin	60.0	41.3 *	38.9 *	44.6	41.2 *
*sitchr*	Siderophore	20.0	33.4 *	31.5	31.7	20.3 ^+^
*ompA*	Protectin	100.0	99.5	100.0	99.7	99.5
*iroN* ^a^	Siderophore	0.0	21.6 *	25.9 *	18.6	10.2 *^,+^
*sitep*	Siderophore	1.7	13.2 *	7.4	10.2	4.3 ^+^
*vat*	Toxin	33.3	22.9	24.1	25.1	15.5 *^,+^
*tsh* ^a^	Adhesin	1.7	7.0	18.5 *	8.1	3.2 ^+^
*iucD* ^a^	Siderophore	8.3	8.6	5.6	8.1	3.2 ^+^
*cvi/cva* ^a^	Protectin	0.0	13.8 *	1.9	9.3	4.3
*papC*	Adhesin	0.0	9.1 *	0.0	6.0	1.6 ^+^
*iss* ^a^	Protectin	10.0	19.7	1.9	15.3	2.1 *^,+^
*EAST1*	Toxin	50.0	24.9 *	29.6 *	30.5	31.6 *
*cnf1/2*	Toxin	0.0	7.3	0.0	4.8	0.0 ^+^
*iutA* ^a^	Siderophore	6.7	8.9	7.4	8.4	3.2 ^+^
*mat*	Adhesin	93.3	74.8 *	85.2	80.2	78.1 *
*fyuA*	Siderophore	23.3	37.2 *	40.7	35.9	20.3 ^+^
*sat*	Toxin	13.3	0.0 *	0.0 *	2.4	0.0 *
*malX*	Miscellaneous	33.3	22.3	25.9	25.1	17.1 *^,+^
*csgA*	Adhesin	95.0	73.4 *	90.7	80.5	89.3 ^+^
*fimC*	Adhesin	100.0	92.7	96.3	94.6	96.8
*irp2*	Siderophore	31.7	34.9	33.3	34.1	20.3 ^+^
*ireA*	Siderophore	6.7	3.9	5.6	4.8	12.8 ^+^
*tia*	Invasin	1.7	2.0	18.5 *	4.8	15.0 *^,+^
*chuA*	Siderophore	23.3	46.9 *	63.0 *	45.8	41.7 *

^a^ Genes associated with large plasmids, like pAPEC-O2-ColV [NC_007675], pTJ100 [AY553855] and pAPEC-O1-ColBM [DQ381420]; * *p* < 0.05 when compared with occurrence percentage in cormorant colony-1; ^+^ *p* < 0.05 when occurrence compared between total birds and wild mammals.

## Data Availability

The data presented in this study is available in the manuscript and its Supplementary Material.

## Supplementary Materials

The following are available online at https://www.mdpi.com/2076-2607/9/2/372/s1, Table S1: Antimicrobial susceptibility testing of *E. coli* isolated from cormorants.
